# Socioeconomic disparities in health outcomes in the United States in the late 2010s: results from four national population-based studies

**DOI:** 10.1186/s13690-023-01026-1

**Published:** 2023-02-04

**Authors:** Yeonwoo Kim, Christian Vazquez, Catherine Cubbin

**Affiliations:** 1 Department of Kinesiology, University of Texas at Arlington, Arlington, TX USA; 2 School of Social Work, University of Texas at Arlington, Arlington, TX USA; 3 Steve Hicks School of Social Work, University of Texas at Austin, Austin, TX USA

**Keywords:** Socioeconomic disparities, Health disparities, Respondent-rated health

## Abstract

**Background:**

Despite the importance of monitoring health disparities by multiple socioeconomic categories, there have been no recent updates on the prevalence of general health indicators by socioeconomic categories. The present study aims to update the prevalence estimates of health indicators by education and income categories across three age groups (children, young and middle-aged adults, and older adults) in the late 2010s by using four nationally representative data sources. We also examine socioeconomic differences in health by race/ethnicity subgroups.

**Methods:**

Data were obtained from four nationally representative data sources from the U.S.: The National Health Interview Survey (2015–2018); the National Health and Nutrition Examination Survey, NHANES (2017–2020); the Behavioral Risk Factor Surveillance System (2016–2020); and the Health & Retirement Study (2016). Respondent-rated health and obesity were selected as the health indicators of interest. Socioeconomic factors included percentages of the federal poverty level and years of educational attainment. We conducted logistic regression analyses to calculate adjusted prevalence rates of respondent-rated (or measured, in the case of obesity in NHANES) poor health and obesity by income and education categories after controlling for sociodemographic characteristics. The complex sampling designs were accounted for in all analyses.

**Results:**

Prevalence rates across racial/ethnic groups and age groups demonstrated clear and consistent socioeconomic gradients in respondent-rated poor health, with the highest rates among those in the lowest income and education categories, and decreased rates as income and education levels increased. On the other hand, there were less evident socioeconomic differences in obesity rates across all data sources, racial/ethnic groups, and age groups.

**Conclusions:**

Our results confirmed earlier, persistent evidence indicating socioeconomic disparities in respondent-rated poor health across all age and race/ethnicity groups by using four nationally representative datasets. In comparison to a decade earlier, socioeconomic disparities in poor health appeared to shrink while they emerged or increased for obesity. The results suggest an urgent need for action to alleviate pervasive health disparities by socioeconomic status. Further research is needed to investigate potentially modifiable factors underlying socioeconomic disparities in health, which may help design targeted health promotion programs.

**Supplementary Information:**

The online version contains supplementary material available at 10.1186/s13690-023-01026-1.

## Background

It is a public health duty to continually monitor socioeconomic disparities in health in the United States (U.S.) and globally. An investigation of socioeconomic patterns in health provides not only the information on the overall burden of health disparities on society [1] but also background data about health status of the most socioeconomically disadvantaged individuals and middle-class individuals in comparison to that of all others. This information is necessary for policy makers and practitioners to develop targeted public health interventions that promote healthy status of the socioeconomically disadvantaged [1].

Since the 1970s, patterns in the health of Americans have faced significant shifts, such as declines in smoking and increases in obesity [2, 3]. Once these shifts were identified and patterns were established, researchers shifted their focus towards understanding sociodemographic differences in health to discover socioeconomic disparities. However, since the turn of the century, only a few studies [1, 4–13] have estimated the adjusted prevalence of adverse health outcomes by multiple socioeconomic categories, and most studies are now more than a decade old. Furthermore, within the limited literature, even less has gone further to include the intersections of age, race/ethnicity, and socioeconomic status (SES), although socioeconomic disparities in health are highly entangled with race/ethnicity in the U.S. For example, although the National Center for Health Statistics within the Centers for Disease Control and Prevention [9] produces a critically important annual report of crude prevalence rates of health status by socioeconomic categories, they do so without also considering differences in health disparities across racial/ethnic groups.

As socioeconomic inequality has increased in recent years [14, 15], an update on recent socioeconomic disparities in health is especially crucial. In particular, we have witnessed substantial macroeconomic fluctuations during the last decade, such as the Great Recession that led to an unprecedented decline in national gross domestic product and a sharp increase in unemployment [16, 17] and disproportionately affected communities with low socioeconomic means [18–21]. At the same time, the Affordable Care Act was passed which could potentially alleviate some socioeconomic or racial/ethnic health disparities through increased access to health care. Furthermore, comparison of health disparities across different data sources is needed because it can provide researchers with clear insights into health disparities in our society. To date, very few studies have illustrated estimates of the population’s health levels by SES categories across different data sources during the same time periods using the same health measures [22, 23]. Those studies showed the challenge of capturing socioeconomic disparities in health when only one data source is used. As the studies were conducted a decade or more ago [22, 23], the current extent and patterns of health disparities need to be investigated with more recent data.

Using four nationally representative data sources from the U.S., the present study aims to update the prevalence estimates of key health indicators by education and income in the late 2010s. We chose respondent-rated health and obesity to represent general health status and health risk because literature shows that these indicators are associated with a higher risk of mortality and a number of other health outcomes [24, 25] and these indicators are recurrent in past prevalence studies [1, 4, 5]. The four data sources that we used include: the National Health Interview Survey (NHIS), 2015–2018 [26]; the National Health and Nutrition Examination Survey (NHANES), 2017-March 2020 [27]; the Behavioral Risk Factor Surveillance System (BRFSS), 2016–2020 [28]; and the Health & Retirement Study (HRS), 2016 [29]. Each data source has a unique strength. NHIS has a large Asian sample (4,378 children, 12,014 middle-aged adults, and 2,716 older adults in our analytic sample), a group that has been understudied in past literature due to the small sample size. NHANES has body mass index information objectively recorded by a trained technician, whereas the other data sources have respondent-rated body mass index information. BRFSS has a very large sample size, and HRS asks detailed income and financial questions to older adults. Using the four data sources allows for comparisons of socioeconomic disparities across databases when using the same health measures captured during the same time period. We also examine socioeconomic differences in these two health indicators by racial/ethnic subgroups because socioeconomic disparities in health are highly entangled with race/ethnicity in the U.S. and there is a disproportionate SES impact on racial/ethnic minorities [30, 31].

## Methods

### Data

We used data from four nationally representative sources to examine and identify robust patterns of socioeconomic disparities in two key health indicators: respondent-rated health and obesity: 1) NHIS, 2) NHANES, 3) BRFSS, and 4) HRS. Details about the design and sampling of each data source have been described elsewhere [26–29]. Table 1 presents a summary of the data sources, sample characteristics, and measures of interest. For NHIS, NHANES, and BRFSS, multiple years of data were pooled together (NHIS: 2015–2018, NHANES: 2017-March 2020, BRFSS: 2016–2020). For HRS, we used 2016 data to match the same time period as the other three sources. We did not include NHIS data past 2018 because there was a sampling design change in 2019 and pooling pre-2019 data with data from 2019 and later is not advised, per the NHIS analytic guidelines [26]. Estimates from pooled analyses are interpreted as an estimate of the average over the time interval of the pooled data [26–28].

**Table 1:** Summary of data sources, sample, measures of socioeconomic status (SES), and health indicators used to examine income and education disparities in health

Data source (Place, Years)	Age groups (Age; Sample size)	Racial/ethnic groups (Sample size)	Measures of SES	Health indicators
National Health Interview Survey (US, 2015–2018)	Children (2–17 y; *n* = 75,111), Young and middle-aged adults (25–64 y; *n* = 181,447), Older adults (≥ 65 y; *n* = 57,753)	Non-Hispanic Blacks (Children: 10,345, Young and middle-aged adults: 21,235, Older adults: 5,457), Hispanic (Children: 19,462, Young and middle-aged adults: 31,150, Older adults: 4,566), Non-Hispanic White (Children: 39,438, Young and middle-aged adults: 114,331, Older adults: 44,461), Non-Hispanic Asian (Children: 4,378, Young and middle-aged adults: 12,014, Older adults: 2,716)	Family income as a percentage of federal poverty level (FPL), educational attainment (for child indicators, head of household; for adult indicators, individual)	Respondent-rated poor health: ‘‘poor,’’ ‘‘fair,’’ or ‘‘good’’ health vs ‘‘very good’’ or ‘‘excellent’’ health Obesity: respondent-assessed body mass index ≥ 30 kg/m^2^
National Health and Nutrition Examination Survey (US, 2017-March 2020)	Children (2–17 y; *n* = 4,292), Young and middle-aged adults (25–64 y; *n* = 5,233), Older adults (≥ 65 y; *n* = 2,022)	Non-Hispanic Black (Children: 1,122, Young and middle-aged adults: 1,434, Older adults: 462), Hispanic (Children: 964, Young and middle-aged adults: 1,167), Non-Hispanic White (Children: 1,434, Young and middle-aged adults: 1,623, Older adults: 1,064)	Family income as a percentage of FPL, educational attainment (for adult indicators only)	Respondent-rated poor health: ‘‘poor,’’ ‘‘fair,’’ or ‘‘good’’ health vs ‘‘very good’’ or ‘‘excellent’’ health Obesity: objectively measured body mass index ≥ 30 kg/m^2^
Behavioral Risk Factor Surveillance System (US, 2016–2020)	Young and middle-aged adults (25–64 y; *n* = 424,595), Older adults (≥ 65 y; *n* = 268,949)	Non-Hispanic Black (Young and middle-aged adults: 34,951, Older adults: 16,296), Hispanic (Young and middle-aged adults: 44,542, Older adults: 10,344), Non-Hispanic Asian (Young and middle-aged adults: 302,223, Older adults: 224,734), Non-Hispanic White (Young and middle-aged adults: 11,475, Older adults: 3,237)	Educational attainment	Respondent-rated poor health: ‘‘poor,’’ ‘‘fair,’’ or ‘‘good’’ health vs ‘‘very good’’ or ‘‘excellent’’ health Obesity: respondent-assessed body mass index ≥ 30 kg/m^2^
Health and Retirement Study (US, 2016)	Older adults (age ≥ 65 y; *n* = 9,539)	Non-Hispanic Black/African American (*n* = 1,589), Hispanic (*n* = 1,076), Non-Hispanic White/Caucasian (*n* = 6,874)	Household income as a percentage of FPL, Educational attainment	Respondent-rated poor health: “poor,” “fair,” or “good,” health vs “very good,” or “excellent” health Obesity: respondent-assessed body mass index ≥ 30 kg/m^2^

### Measures

Respondent-rated health and obesity were selected as the health indicators of interest because of their ubiquity within nationally representative data sources and across data collection time points. Additionally, both indicators have been well-documented over time allowing for comparisons against previous studies and future studies to determine changes in the health of the population with these indicators as proxies. Respondent-rated health is generally asked in the following form, “Would you say that in general your health is…excellent, very good, good, fair, poor.” This was dichotomized as good health (excellent/very good, 0) and poor health (good/fair/poor, 1) based on previous literature (1). Parent/guardian respondents answered this question on behalf of children under the age of sixteen. Obesity was defined as a body mass index ≥ 30 kg/m^2^. For all data sources, except NHANES, body mass index was based on self-reported height and weight. Parent/guardian respondents answered this question on behalf of children under the age of sixteen. For NHANES, all body measurements were objectively recorded by a trained technician. This variable was dichotomized as obese (1) and not obese (0).

Socioeconomic factors included household income as a percentage of the federal poverty level (based on the survey year; < 100%, 100–199%, 200–299%, and 300+%) and years of educational attainment (< high school graduate, high school graduate, some college, college graduate). In addition, we utilized racial/ethnic subgroups for which sample sizes were sufficient. For NHIS and BRFSS we used four subgroups: non-Hispanic Black/African American, Hispanic, non-Hispanic White, and non-Hispanic Asian. For NHANES and HRS, we used three subgroups: non-Hispanic Black/African American, Hispanic, and non-Hispanic White. We also created age subgroups within the adult samples of the NHIS, NHANES, and BRFSS. The adult samples were grouped into two groups: young and middle-aged adults (25–64 years) and older adults (65 years and older).

### Analysis

We conducted logistic regression analyses to calculate adjusted prevalence rates of respondent-rated poor health and obesity with 95% confidence intervals by income and education categories, adjusted for age, sex, race/ethnicity, and education or income. For racial/ethnic subgroups, adjusted rates were calculated after controlling for age, sex, and education or income. This was the case for all analyses except: BRFSS in which income was missing for over 10% of respondents and otherwise grouped into categories that preclude accurate federal poverty level estimates at higher income levels; and NHANES in which data was missing for parents’ educational attainment for children due to changes in the survey design in 2019 and 2020. Thus, models utilizing BRFSS data only examined patterns by educational attainment, and models utilizing NHANES data, for children, only examined patterns by household income. Trend tests were performed, which tested whether the slope, or socioeconomic gradient in health, differed from zero. Trend tests included the same controls that were used for the prevalence estimates. All data sources utilized a complex sampling design; thus, prevalence rates were calculated by including sample weights and design-related statements were used to produce valid standard errors. For the pooled data sets, a new sample weight was computed by adding the weights and dividing them by the number of years included. This was done per the analytic guidelines for each data source [26–28]. An alpha of 0.05 was used to test significance. All analyses were conducted in STATA 17 [32].

## Results

### Respondent-rated health

#### Children

**Fig. 1:**
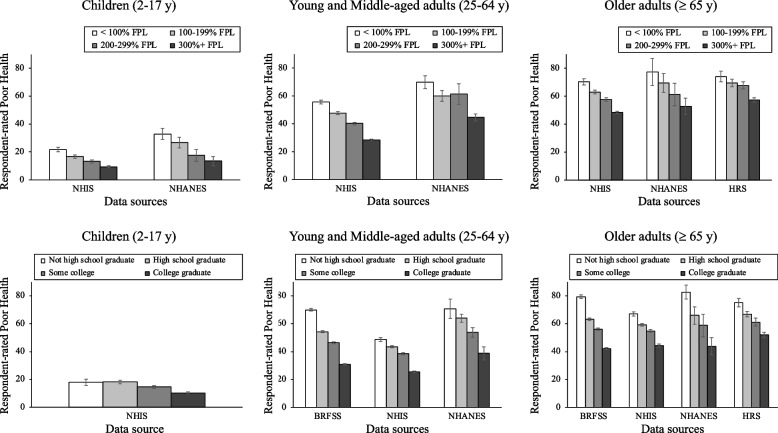
Income and education disparities in respondent-rated health across data sources Note. FPL = federal poverty level. Source. Our data from the National Health Interview Study (NHIS) was collected in the US in 2015–2018. Data from the National Health and Nutrition Examination Survey (NHANES) was collected in the US in 2017-March 2020. Data from the Health and Retirement Study (HRS) was collected in the US in 2016. Data from the Behavioral Risk Factor Surveillance System (BRFSS) was collected in the US in 2016–2020

The data sources for the income models for children include NHIS and NHANES. For the education models, only NHIS was used. As shown in Fig. 1, children from the lowest-income or least-educated families had the highest rate of poor health, and rates of poor health decreased at each higher income or education level. When examining socioeconomic disparities in health by race/ethnicity, the income and education gradients were observed among all racial/ethnic groups, except for Black children from the NHANES sample (see Appendix Table 1) and the Asian sample from the NHIS (see Appendix Table 2). In addition, for both the income and education models, the Black and Hispanic samples had higher prevalence rates of respondent-rated poor health across all socioeconomic categories than the Asian and White samples (see Appendix Tables 1–2).

### Young and Middle-aged adults

The data sources for the income models include NHIS and NHANES, with the addition of BRFSS for the education models. The results showed the highest rate of poor health among the lowest-income or least-educated individuals and clear stepwise patterns by income and education categories (see Fig. 1 and Appendix Tables 1–2). The socioeconomic gradients were also observed among all racial/ethnic groups (see Appendix Figs. 1–4 and Appendix Tables 1–2). In addition, patterns often showed that the Black and Hispanic samples had higher prevalence rates of respondent-rated poor health across all socioeconomic categories than the Asian and White samples (see Appendix Tables 1–2).

### Older adults

The data sources for the income models include HRS, NHIS, and NHANES, with the addition of BRFSS for the education models. The results showed the highest rate of poor health in the lowest-income or least-educated individuals and significant health gaps between those with middle SES category and the highest SES category among the total sample (see Fig. 1). In addition, the socioeconomic gradients were observed across all racial/ethnic subgroups except for the Black sample from NHANES (see Appendix Figs. 1–4 and Appendix Tables 1–2). Furthermore, in the income and education models across all data sources, the Black and Hispanic samples often had higher prevalence rates of respondent-rated poor health across all socioeconomic categories than the Asian and White samples (see Appendix Tables 1–2).

## Obesity

### Children

**Fig. 2:**
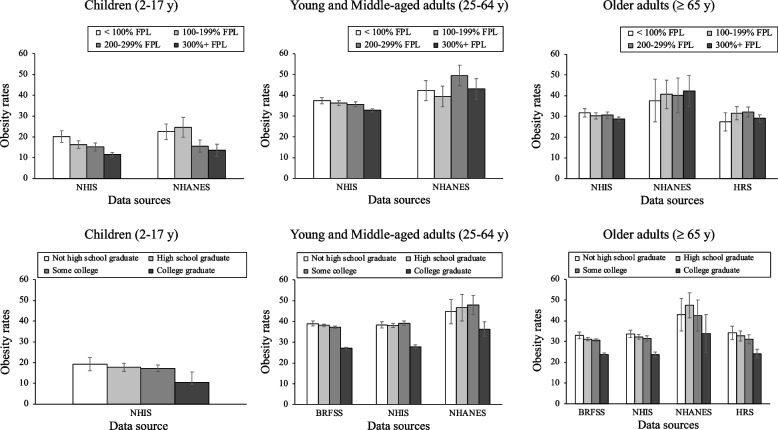
Income and education disparities in obesity across data sources Note. FPL = federal poverty level. Source. Our data from the National Health Interview Study (NHIS) was collected in the US in 2015–2018. Data from the National Health and Nutrition Examination Survey (NHANES) was collected in the US in 2017-March 2020. Data from the Health and Retirement Study (HRS) was collected in the US in 2016. Data from the Behavioral Risk Factor Surveillance System (BRFSS) was collected in the US in 2016–2020

The data sources for the income models for children include NHIS and NHANES. For the education models, only NHIS was used. In the income and education models, rates of obesity appeared to decrease as income and education went up in the NHIS data but not in the NHANES data (see Fig. 2). In addition, the patterns varied by racial/ethnic group (see Appendix Figs. 5–8 and Appendix Tables 3–4). Specifically, while the income and education gradients were observed in the White sample, there were inconclusive patterns for Asian, Black and Hispanic samples depending on data sources, with some rates showing increases as income or education went up and others showing decreases (see Appendix Figs. 5–8 and Appendix Tables 3–4). In addition, in the income models, the Black and Hispanic samples shared the highest obesity rates across all income categories (see Appendix Table 3). In the education models, the Black sample often had the highest obesity rates across all education categories (see Appendix Table 4).

### Young and Middle-aged adults

The data sources for the income models include NHIS and NHANES, with the addition of BRFSS for the education models. Trend patterns were inconclusive in the income models. The income gradient was observed only in the models with the NHIS, not in the models with the NHANES (see Fig. 2 and Appendix Table 3). In the racial/ethnic subgroup analysis of the income models, the income gradient was evident for Hispanic and White adults, but not for Black and Asian adults (see Appendix Figs. 1–4 and Appendix Table 3). On the other hand, in the education models, the education gradient was consistently observed as shown in Fig. 2. When examining the education gradient by race/ethnicity, the education gradient was consistently observed in the White sample across all data sources, but the Black, Hispanic, and Asian samples showed different trend patterns by data sources (see Appendix Figs. 5–8 and Appendix Table 4). Furthermore, in all the income and education models, the Black sample had the highest obesity rate across all income/education categories (see Appendix Tables 3–4).

### Older adults

The data sources for the income models include HRS, NHIS, and NHANES, with the addition of BRFSS for the education models. In the income models, there were inconclusive trend patterns as shown in Fig. 2. Specifically, the income gradient was observed only in the NHIS, but not in the NHANES (see Appendix Table 3). In the racial/ethnic subgroup analysis of the income models, there were no significant income gradients except for the Hispanic sample in NHIS (see Appendix Figs. 5–8 and Appendix Table 3). In the education models, the education gradient was notable in the total sample, as shown in Fig. 2 and Appendix Table 4. When examining the education gradient by race/ethnicity, the education gradient was consistently observed in the White sample across data sources. For the Hispanic and Black samples, there were no significant education gradient. In the income and education models, the Black sample had the highest obesity rate across all income categories.

### Comparing patterns across data sources

In the respondent-rated health analyses (see Fig. 1), the NHANES data tended to produce higher prevalence rates across all groups and socioeconomic factors compared to the three other data sources. The NHIS produced the lowest prevalence rates compared to all three other data sources. The differences across data sources were particularly notable for the most disadvantaged groups as they were higher and more varied across sources (i.e., < 100% FPL and not high school graduate); whereas the prevalence rates for the other three groups within each category tended to cluster around the same levels.

In the obesity analyses (see Fig. 2), the NHANES data again produced higher prevalence rates across all groups and socioeconomic factors compared to all three other data sources. The NHIS, BRFSS and HRS produced similar prevalence rates when compared to each other. Unlike the respondent-rated health analyses, the differences across data sources were more notable for the most advantaged groups than the most disadvantaged group. For all four data sources, the prevalence rates across the three less advantaged groups clustered around similar levels; whereas the prevalence rates for the most advantaged groups (i.e., 300+% FPL and college graduate) tended to be at notably lower and varying levels across sources.

## Discussion

Despite perpetuating socioeconomic differences in health, there have been no recent updates on prevalence rates of general health indicators by SES aside from the NCHS report, which does not delve deeper into the intersections of race/ethnicity, age, and income or education [9]. A lack of knowledge about the recent prevalence of adverse health outcomes by socioeconomic categories limits us to an understanding of socioeconomic disparities in health during the last decade. This study addressed this limitation of knowledge by describing recent patterns of socioeconomic differences in health indicators among children, young and middle-aged adults, and older adults and comparing the pattern and extent of socioeconomic disparities in health across different data sources. We first estimated socioeconomic differences in prevalence rates of respondent-rated poor health and obesity by age groups and then by age and race/ethnicity groups in the late 2010s given that socioeconomic status is strongly related to race/ethnicity in the U.S. [30, 31].

Our findings revealed clear and consistent socioeconomic gradients in respondent-rated poor health among the three age groups and all 12 age and race/ethnicity groups, after adjusting for sociodemographic characteristics (i.e., age, sex, race/ethnicity, education, or income). Specifically, prevalence rates of poor health were often the highest among those in the lowest income and education categories regardless of age cohort and race/ethnicity. We also observed a large health gap between those in the middle levels of income and education and those in the highest levels. The socioeconomic gradients in respondent-rated poor health align with earlier evidence indicating socioeconomic gradients in health in the U.S. [1, 4–8, 10–13]. Of note, the extent of socioeconomic disparities in respondent-rated poor health appeared to decrease compared to that in the 2000s estimated by Braveman et al. [1]. For example, when using the NHANES, the percentage of non-high school graduate adults with poor health decreased from 77.4% in 1994–2004 to 70.7% in 2017–2020 while the percentage of college graduate adults with poor health increased from 30.0% to 38.8% during the same observation period.

On the other hand, obesity rates showed less evident socioeconomic differences than respondent-rated poor health. Specifically, in the education models, consistent educational differences in obesity were found only among the total sample and Whites, but not among Asians, Blacks, and Hispanics. In particular, when comparing it to statistics in 2005–2007 [1], educational differences in obesity became more pronounced—while BRFSS showed no educational differences in 2005–2007, significant educational differences in obesity were observed in 2016–2020. Income differences in obesity were inconsistent across all age and racial/ethnic groups. The results indicate that different socioeconomic patterns in the two health indicators are possibly due to different underlying mechanisms of respondent-rated health and obesity. While respondent-rated health status represents an individual’s overall health status and well-being, obesity is specifically related to metabolic and cardiovascular risk. Socioeconomic status may be more impactful for certain health outcomes. The explanation is in line with previous research reporting a less clear socioeconomic gradient in body mass index, scores for healthy eating, and prevalence rates of diabetes [1, 6]. Future research is needed to elaborate underlying mechanisms of different socioeconomic patterns in various health indicators, which helps policy makers and practitioners design more targeted programs to decrease socioeconomic gaps in health.

The overall picture of socioeconomic disparities in health was found to be more complicated by considering age and race/ethnicity. When comparing prevalence rates of respondent-rated poor health between the lowest and highest income and education categories, Blacks and Hispanics appeared to have the largest socioeconomic difference during childhood. Given Blacks and Hispanics often had the highest rates of respondent-rated poor health and obesity in the same income and education levels, regardless of age groups, policies and programs designed for Black and Hispanic children from households with low SES may help alleviate health disparities by both SES and race/ethnicity. On the other hand, although Blacks often had worse health outcomes than other racial/ethnic groups, Whites had the largest socioeconomic difference in respondent-rated health for young and middle age adulthood; and for older adulthood, Whites and Hispanics often had the largest socioeconomic difference in respondent-rated health. In other words, for adults, Blacks had worse health outcomes across all SES categories than other racial/ethnic groups, but their socioeconomic differences in health were relatively small. The results imply that policy strategies designed to promote health of adults with low SES may be more likely to target Whites, which may not be effective to tackle racial/ethnic disparities in health. Thus, multiple policy strategies should be adopted to alleviate both racial/ethnic and socioeconomic disparities in adult health. Future research is warranted to investigate underlying mechanisms for racial/ethnic and socioeconomic differences in health across age groups for a better understanding of interactions between age, race/ethnicity, and SES in health disparities.

Looking across the data sources, there are visible differences in prevalence rates of health conditions across data sources when using the same health measures around the same time and controlling for the same factors. Prevalence rates for NHANES were notably higher compared to all three other data sources, and the NHIS produced the lowest prevalence rates of respondent-rated health status. The results are consistent with Nelson et al. [22] who reported differences in prevalence rates of health indicators (e.g., height, weight, and respondent-rated health status) between the NHIS and the BRFSS. Specifically, while Nelson et al. [22] found lower estimates of respondent-rated health in NHIS compared to BRFSS as is with the present study, obesity rates were higher in NHIS than BRFSS, which is the opposite in our study. Nelson et al.’s study is nearly two-decades old, and our findings align with the findings from Pemberton et al.’s study [23] in that that the NHIS estimates tend to be lower than other data sources. Investigating the nuances of differences across data sources is beyond the scope of this study, but differences in estimates possibly result from several methodological differences, such as type and mode of data collection, weighting and representativeness of the sample, question placement, wording, format, use of proxy reporting for youth, and not completely overlapping data collection periods. Future research needs to examine underlying reasons for different estimates of health indicators to understand which data sources may provide the most accurate estimates of the population’s health levels.

This study has several limitations. Our analysis was limited to two health indicators, respondent-rated health status and obesity. Although the measures are widely used and represent general health status [1, 4, 5, 24, 25], using a more comprehensive set of health indicators will allow future studies to examine different mechanisms underlying socioeconomic disparities in various health indicators. This study did not use precisely overlapping years due to various reasons explained in the methods; however, there were no major events (e.g., recessions or pandemics) in the years utilized across data sources. Also, this study did not examine mechanisms of health disparities. Future research needs to compare mechanisms of health disparities by SES and race/ethnicity. Racial/ethnic disparities in health may be related to racial/ethnic discrimination, racial/ethnic segregation, and lack of health-promoting resources in minority neighborhoods that Blacks and Hispanics often experience in their daily life. Further research is warranted to include various potential causes of racial/ethnic and socioeconomic disparities for an investigation of mechanisms of health disparities. Another limitation of this study is that respondent-assessed body mass index was used to operationalize obesity in the NHIS, BRFSS, and HRS. Although the NHANES has body mass index information objectively measured by a trained technician, more replication studies are needed to draw solid conclusions. Lastly, although our study is unique in that Asians, who have been understudied in past literature, are included in our analysis, our study could not include more specific Asian subgroups, Pacific Islanders, American Indians, or Hispanic subpopulations due to a lack of sufficient data.

## Conclusions

Health disparities are a pervasive social issue that requires intervention efforts from government agencies, communities, and researchers. Using four nationally representative data sources collected in the late 2010s, the present study confirmed that socioeconomic disparities in health, especially respondent-rated health, are persistent and pervasive, despite expanding health care access resulting from the Affordable Care Act, emphasizing the fundamental importance of social determinants of health. Our findings may inform future research to explore potentially preventable or modifiable factors underlying health disparities in order to design targeted policies and programs aimed at promoting population health. In addition, as health data collected during the COVID-19 pandemic from nationally representative U.S. samples become available in the future [33], the results may be informative as baseline statistics for future research on changing socioeconomic disparities in health before, during, and after the COVID-19 pandemic.

## Supplementary Information

**Additional file 1.**

## Data Availability

The datasets generated and/or analyzed during the current study are available in each study’s repository: (1) BRFSS repository at https://www.cdc.gov/brfss/annual_data/annual_data.htm, (2) HRS repository at http://hrsonline.isr.umich.edu, (3) NHANES repository at https://wwwn.cdc.gov/nchs/nhanes, (4) NHIS repository at https://www.cdc.gov/nchs/nhis/2019nhis.htm.

## Abbreviations

BRFSS: Behavioral Risk Factor Surveillance System
HRS: Health & Retirement Study
NHANES: National Health and Nutrition Examination Survey
NHIS: National Health Interview Survey
SES: Socioeconomic status
U.S.: United States
