# Supplementary material for: Socioeconomic disparities in health outcomes in the United States in the late 2010s: results from four national population-based studies
Source: Arch Public Health. 2023 Feb 4;81:15. doi: 10.1186/s13690-023-01026-1 (PMC9899106; doi:10.1186/s13690-023-01026-1)
Supplement: Supplementary file 1 — Additional file 1. [file 13690_2023_1026_MOESM1_ESM.pdf]

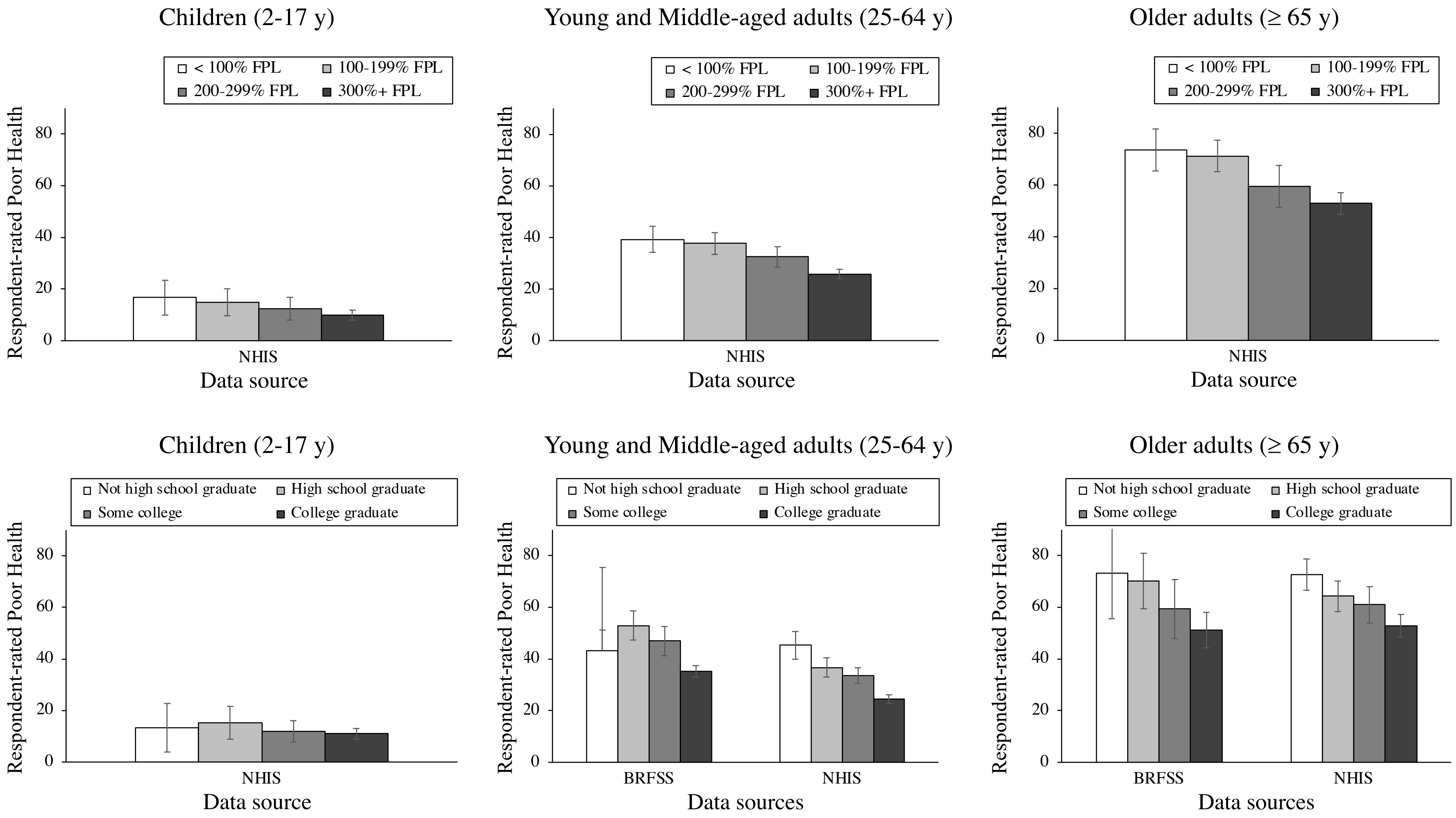

Appendix Figure 1. Income and education disparities in respondent-rated health among non-Hispanic Asians across data sources

Note. FPL = federal poverty level

Source. Our data from the National Health Interview Study (NHIS) was collected in the US in 2015-2018. Data from the Behavioral Risk Factor Surveillance System (BRFSS) was collected in the US in 2016-2020.

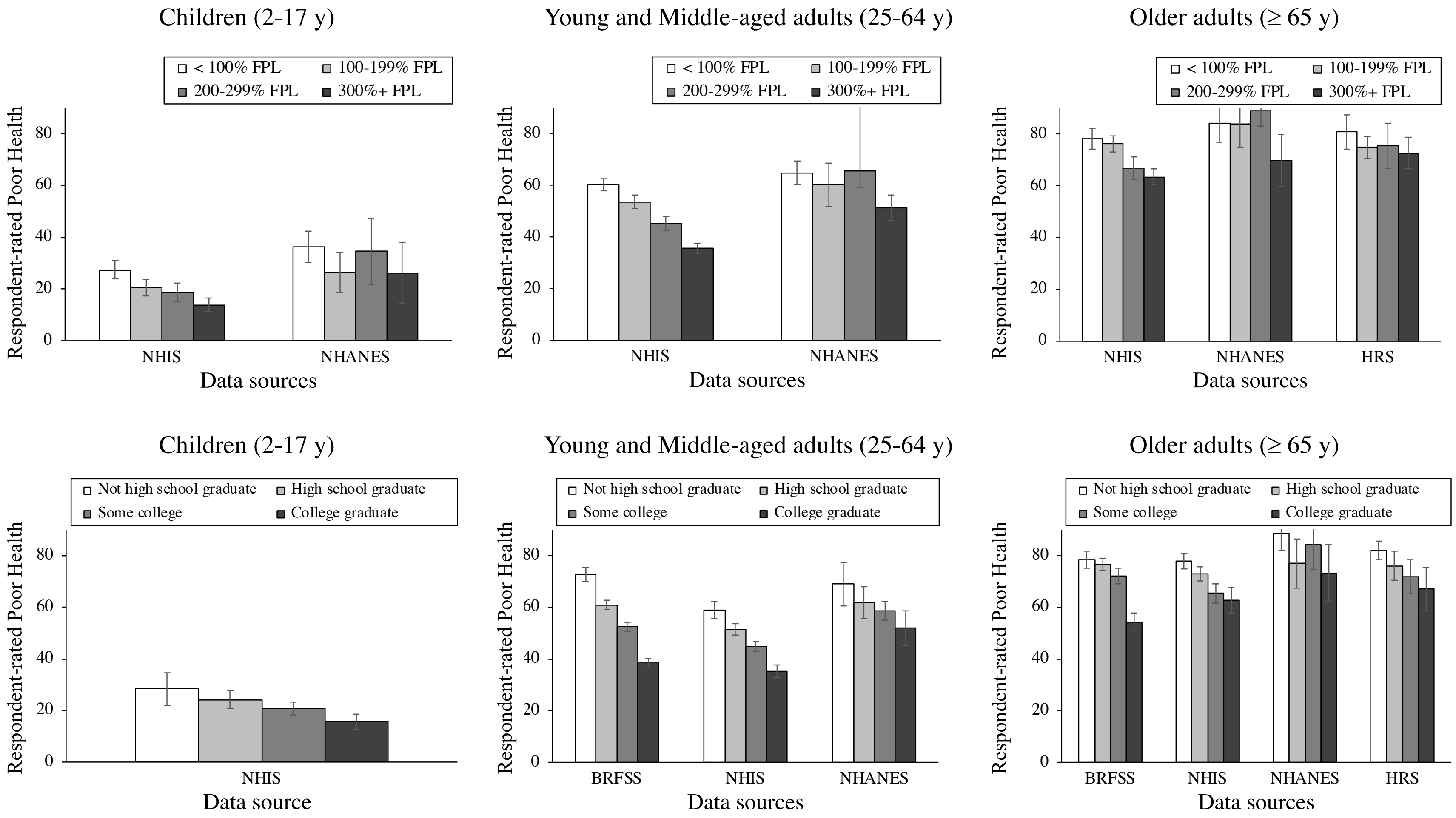

Appendix Figure 2. Income and education disparities in respondent-rated health among non-Hispanic Blacks across data sources

Note. FPL = federal poverty level
Source. Our data from the National Health Interview Study (NHIS) was collected in the US in 2015-2018. Data from the National Health and Nutrition Examination Survey (NHANES) was collected in the US in 2017-March 2020. Data from the Health and

Retirement Study (HRS) was collected in the US in 2016. Data from the Behavioral Risk Factor Surveillance System (BRFSS) was collected in the US in 2016-2020.

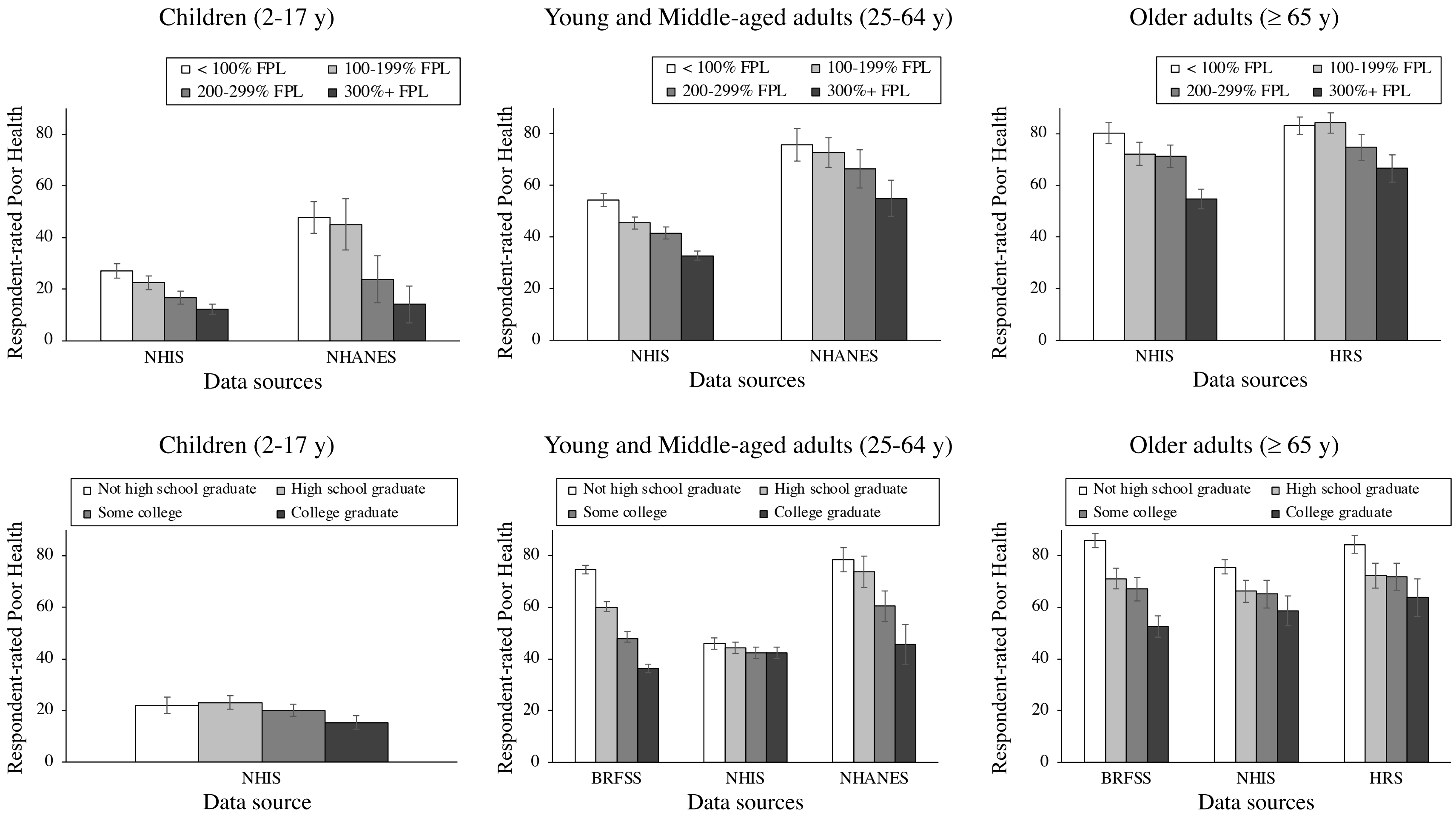

Appendix Figure 3. Income and education disparities in respondent-rated health among Hispanics across data sources

Note. FPL = federal poverty level
Source. Our data from the National Health Interview Study (NHIS) was collected in the US in 2015-2018. Data from the National Health and Nutrition Examination Survey (NHANES) was collected in the US in 2017-March 2020. Data from the Health and

Retirement Study (HRS) was collected in the US in 2016. Data from the Behavioral Risk Factor Surveillance System (BRFSS) was collected in the US in 2016-2020.

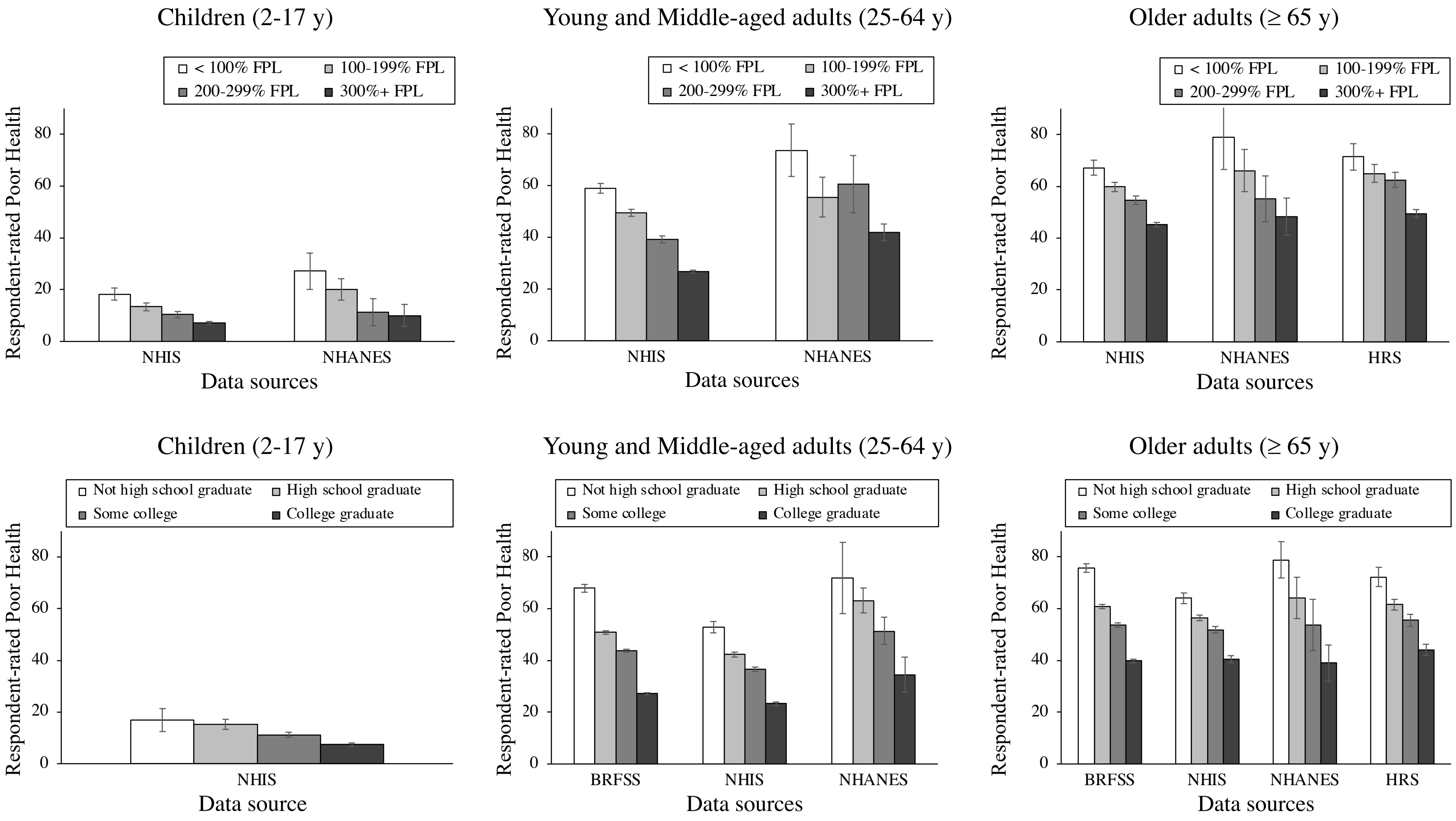

Appendix Figure 4. Income and education disparities in respondent-rated health among non-Hispanic Whites across data sources

Note. FPL = federal poverty level

Source. Our data from the National Health Interview Study (NHIS) was collected in the US in 2015-2018. Data from the National Health and Nutrition Examination Survey (NHANES) was collected in the US in 2017-March 2020. Data from the Health and

Retirement Study (HRS) was collected in the US in 2016. Data from the Behavioral Risk Factor Surveillance System (BRFSS) was collected in the US in 2016-2020.

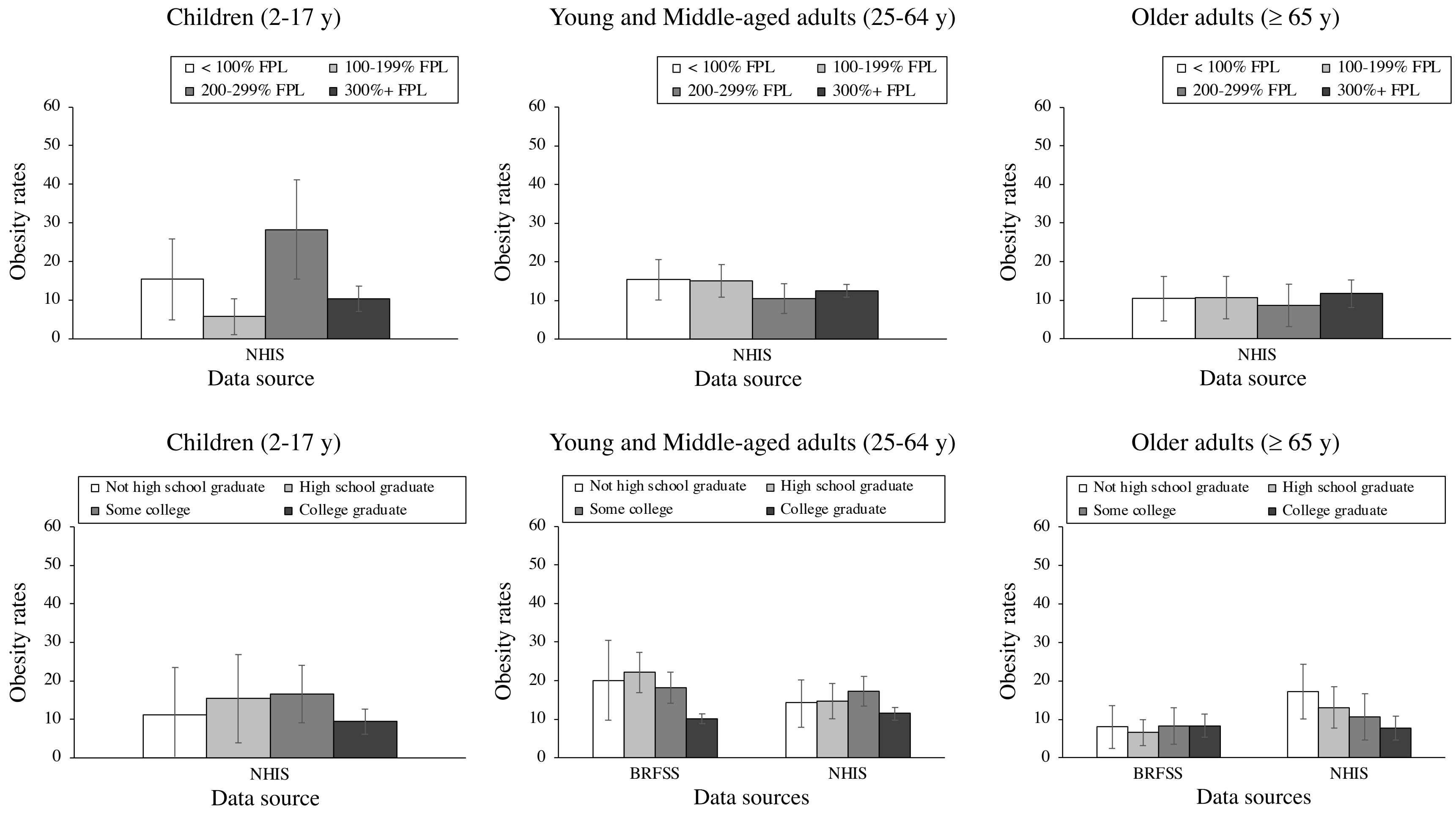

Appendix Figure 5. Income and education disparities in obesity among non-Hispanic Asians across data sources

Note. FPL = federal poverty level

Source. Our data from the National Health Interview Study (NHIS) was collected in the US in 2015-2018. Data from the Behavioral Risk Factor Surveillance System (BRFSS) was collected in the US in 2016-2020.

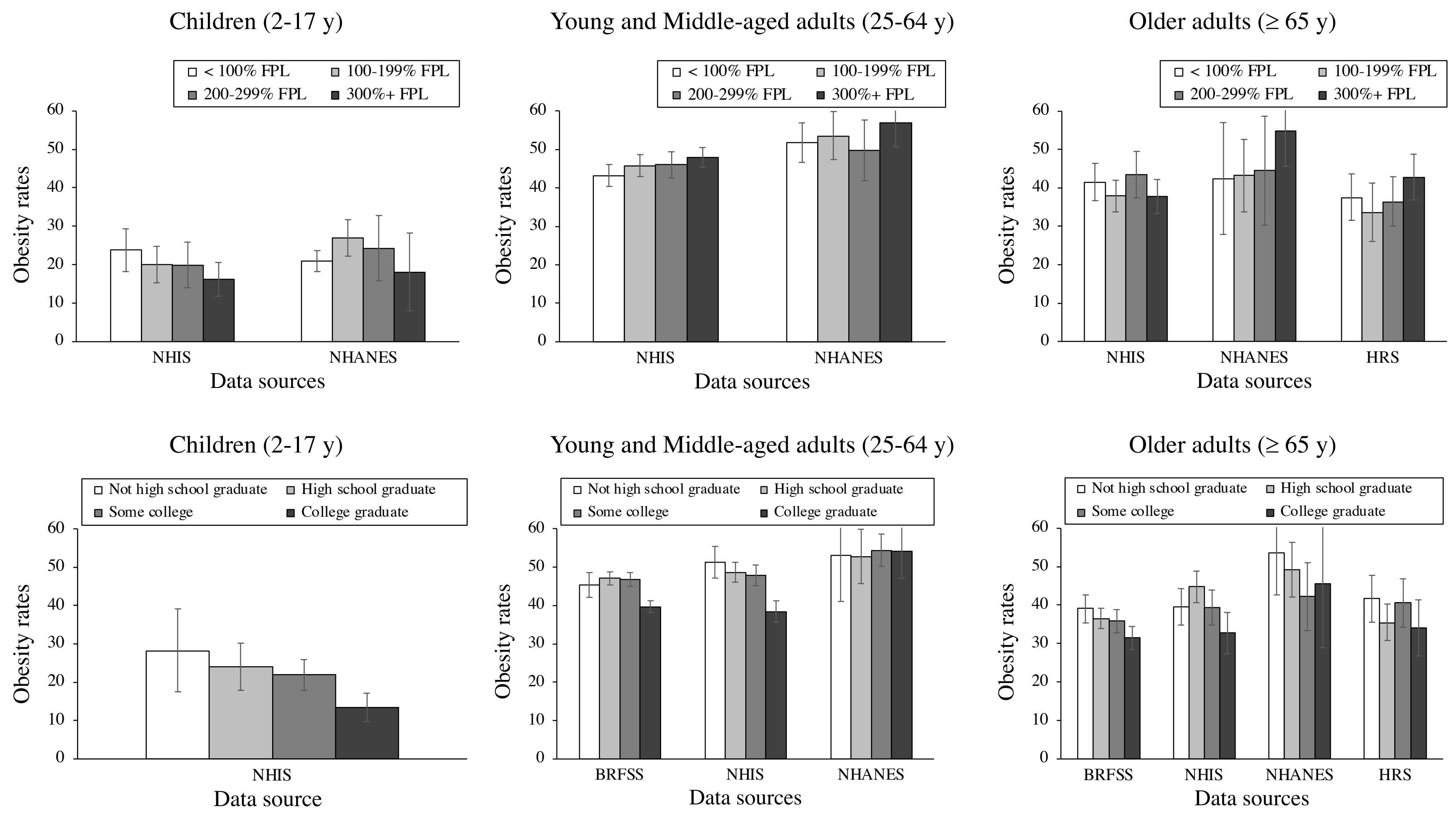

Appendix Figure 6. Income and education disparities in obesity among non-Hispanic Blacks across data sources

Note. FPL = federal poverty level

Source. Our data from the National Health Interview Study (NHIS) was collected in the US in 2015-2018. Data from the National Health and Nutrition Examination Survey (NHANES) was collected in the US in 2017-March 2020. Data from the Health and

Retirement Study (HRS) was collected in the US in 2016. Data from the Behavioral Risk Factor Surveillance System (BRFSS) was collected in the US in 2016-2020.

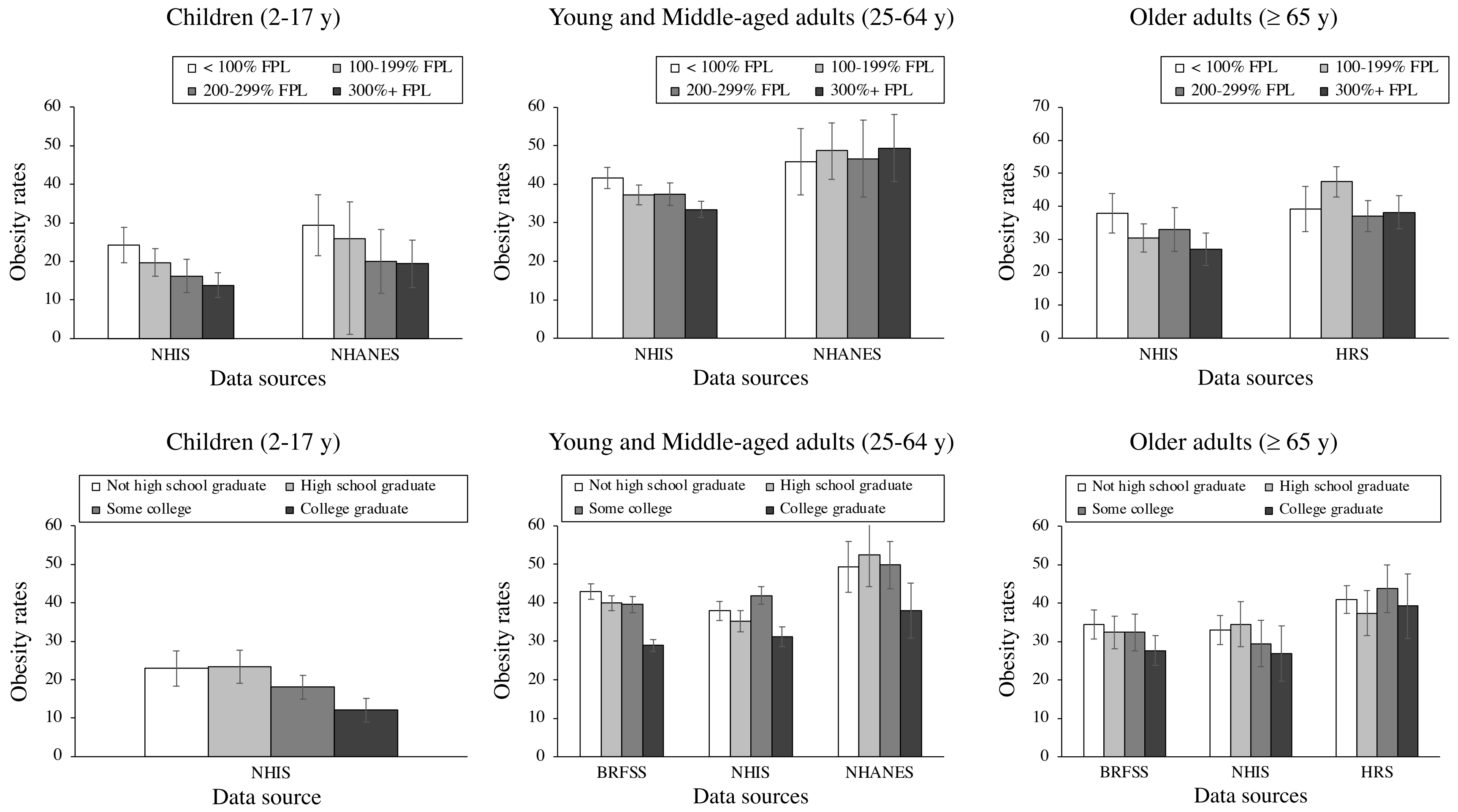

Appendix Figure 7. Income and education disparities in obesity among Hispanics across data sources

Note. FPL = federal poverty level

Source. Our data from the National Health Interview Study (NHIS) was collected in the US in 2015-2018. Data from the National Health and Nutrition Examination Survey (NHANES) was collected in the US in 2017-March 2020. Data from the Health and

Retirement Study (HRS) was collected in the US in 2016. Data from the Behavioral Risk Factor Surveillance System (BRFSS) was collected in the US in 2016-2020.

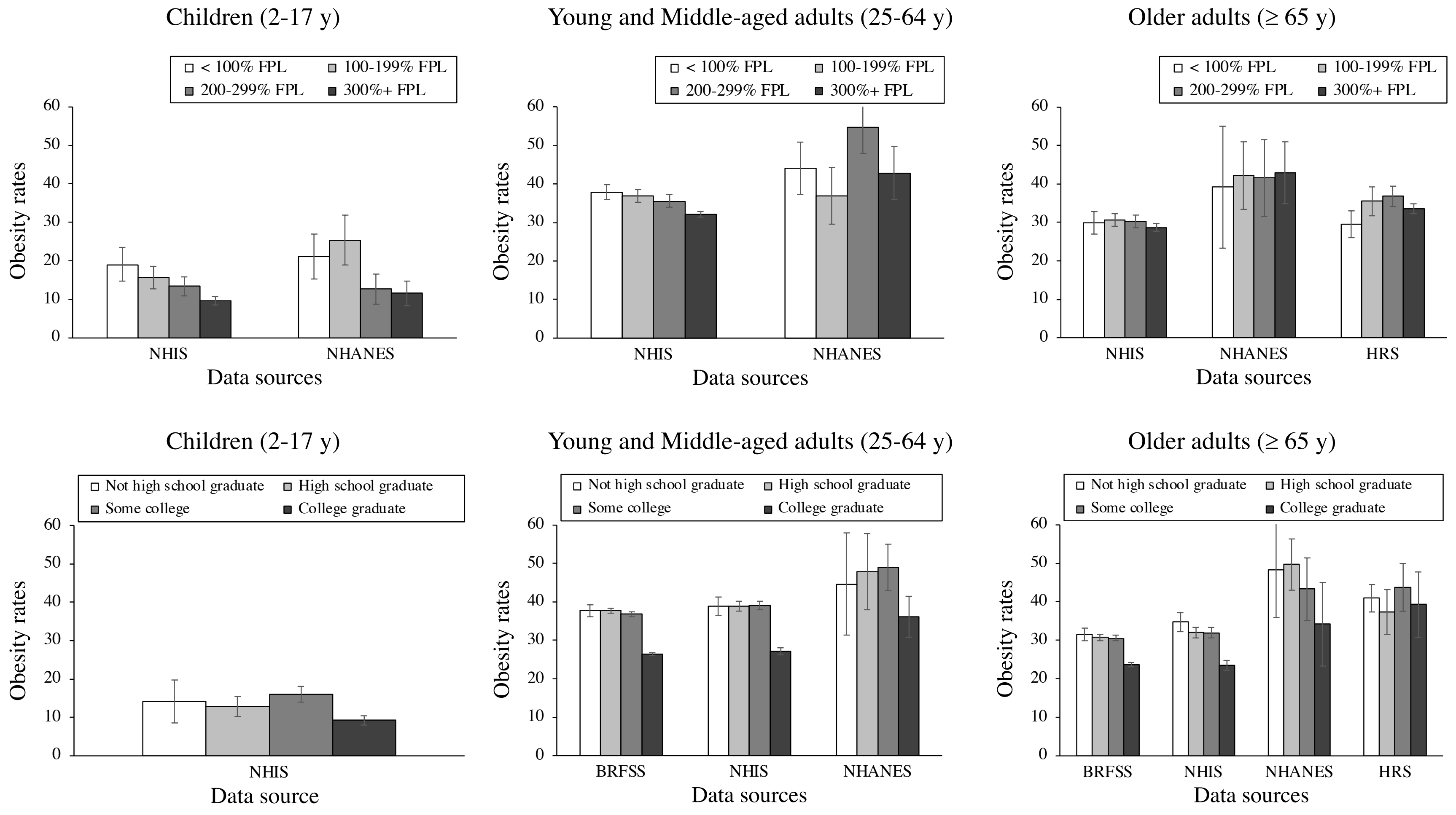

Appendix Figure 8. Income and education disparities in obesity among non-Hispanic Whites across data sources

Note. FPL = federal poverty level
Source. Our data from the National Health Interview Study (NHIS) was collected in the US in 2015-2018. Data from the National Health and Nutrition Examination Survey (NHANES) was collected in the US in 2017-March 2020. Data from the Health and

Retirement Study (HRS) was collected in the US in 2016. Data from the Behavioral Risk Factor Surveillance System (BRFSS) was collected in the US in 2016-2020.

Appendix Table 1. Adjusted rates of respondent-rated poor health by income categories

Data name and population subgroup	Adjusted rates by family income as percentage of FPL[1] < 100% FPL, % (95% CI)	100-199% FPL, % (95% CI)	200-299% FPL, % (95% CI)	≥ 300% FPL, % (95% CI)	Trend, *P*
National Health Interview Survey					
All (2-17 y)	21.7 (20.1, 23.4)	16.7 (15.5, 17.9)	13.2 (12.1, 14.3)	9.4 (8.8, 10.1)	<.001
NH Black (2-17 y)	27.4 (23.9, 31.0)	20.6 (17.5, 23.7)	18.7 (15.1, 22.2)	13.9 (11.3, 16.5)	<.001
Hispanic (2-17 y)	27.2 (24.4, 30.0)	22.5 (19.9, 25.1)	16.7 (14.3, 19.1)	12.2 (10.2, 14.3)	<.001
NH White (2-17 y)	18.3 (15.9, 20.6)	13.4 (11.8, 15.0)	10.5 (9.2, 11.7)	7.2 (6.6, 7.8)	<.001
NH Asian (2-17 y)	16.7 (9.9, 23.5)	15.0 (9.8, 20.2)	12.3 (7.9, 16.8)	10.0 (8.0, 11.9)	.010
All (25-64 y)	55.6 (54.3, 56.9)	47.7 (46.5, 48.8)	40.2 (39.2, 41.2)	28.5 (28.0, 29.1)	<.001
NH Black (25-64 y)	60.2 (57.8, 62.6)	53.5 (50.9, 56.1)	45.2 (42.4, 48.0)	35.7 (33.7, 37.7)	<.001
Hispanic (25-64 y)	54.2 (51.7, 56.7)	45.4 (43.1, 47.8)	41.4 (39.1, 43.8)	32.7 (30.9, 34.5)	<.001
NH White (25-64 y)	58.8 (57.0, 60.7)	49.4 (48.0, 50.8)	39.2 (37.8, 40.6)	26.8 (26.1, 27.4)	<.001
NH Asian (25-64 y)	39.3 (34.2, 44.5)	37.7 (33.4, 41.9)	32.5 (28.5, 36.5)	25.8 (24.1, 27.6)	<.001
All (≥ 65 y)	70.3 (68.1, 72.5)	63.0 (61.6, 64.4)	57.6 (56.3, 59.0)	48.3 (47.5, 49.2)	<.001
NH Black (≥ 65 y)	78.2 (74.2, 82.3)	76.2 (73.1, 79.3)	66.9 (62.6, 71.2)	63.3 (60.6, 66.5)	<.001
Hispanic (≥ 65 y)	80.2 (76.2, 84.3)	72.2 (67.7, 76.7)	71.3 (66.9, 75.7)	54.8 (50.9, 58.6)	<.001
NH White (≥ 65 y)	67.2 (64.4, 70.0)	59.8 (58.1, 61.5)	54.6 (53.1, 56.2)	45.3 (44.3, 46.2)	<.001
NH Asian (≥ 65 y)	73.6 (65.6, 81.7)	71.2 (65.2, 77.3)	59.5 (51.5, 67.6)	52.9 (48.8, 57.1)	<.001
National Health and Nutrition Examination Survey					
All (2-17 y)	32.9 (29.0, 36.8)	26.8 (23.0, 30.6)	17.6 (13.2, 21.9)	13.4 (10.2, 16.7)	<.001
NH Black (2-17 y)	36.4 (30.3, 42.5)	26.4 (18.8, 34.0)	34.6 (21.9, 47.3)	26.3 (14.7, 38.0)	<.001
Hispanic (2-17 y)	47.8 (41.6, 54.0)	45.1 (35.2, 55.0)	23.8 (14.8, 32.9)	14.1 (6.9, 21.2)	<.001
NH White (2-17 y)	27.4 (20.1, 34.2)	20.1 (15.9, 24.3)	11.4 (6.2, 16.5)	10.0 (5.8, 14.3)	<.001
All (25-64 y)	69.8 (65.1, 74.5)	60.1 (56.3, 63.9)	61.4 (54.1, 68.7)	44.6 (42.0, 47.1)	<.001
NH Black (25-64 y)	64.7 (60.2, 69.2)	60.3 (51.9, 68.6)	65.5 (59.1, 71.9)	51.2 (46.2, 56.3)	.001
Hispanic (25-64 y)	75.7 (69.4, 82.0)	72.6 (66.9, 78.3)	66.3 (58.8, 73.8)	54.9 (47.9, 61.9)	<.001
NH White (25-64 y)	73.5 (63.4, 83.7)	55.5 (47.8, 63.3)	60.6 (49.5, 71.6)	41.9 (38.6, 45.2)	<.001
All (≥ 65 y)	77.4 (67.6, 87.1)	69.5 (62.8, 76.2)	61.3 (53.3, 69.2)	52.8 (46.9, 58.6)	<.001
NH Black (≥ 65 y)	84.1 (76.8, 91.3)	83.9 (75.0, 92.7)	89.1 (83.1, 95.2)	69.8 (59.7, 79.8)	.040
NH White (≥ 65 y)	79.0 (66.5, 91.5)	66.1 (57.9, 74.2)	55.3 (46.5, 64.1)	48.3 (41.0, 55.6)	<.001
Health & Retirement Study (≥ 65 y)					

All	74.0 (70.3, 77.8)	69.6 (66.8, 72.3)	67.7 (65.1, 70.3)	57.3 (55.7, 59.0)	<.001
NH Black	80.9 (74.2, 87.5)	74.9 (70.7, 79.1)	75.5 (66.7, 84.2)	72.6 (66.5, 78.7)	.116
Hispanic	83.2 (79.8, 86.5)	84.2 (80.2, 88.1)	74.7 (69.6, 79.7)	66.6 (61.2, 71.9)	<.001
NH White	71.4 (66.3, 76.4)	65.0 (61.5, 68.5)	62.5 (59.5, 65.4)	49.5 (48.0, 51.0)	<.001

Note. NH indicates non-Hispanic. CI indicates confidence interval.

Source. Our data from the National Health Interview Study was collected in the US in 2015-2018. Data from the National Health and Nutrition Examination Survey was collected in the US in 2017-March 2020. Data from the Health and Retirement Study was collected in the US in 2016.

[1] Age, sex, race/ethnicity, and education level were adjusted in the full sample. In race/ethnicity-stratified sample, age, sex, and education level were adjusted. As an exception, education was not adjusted in the National Health and Nutrition Examination Survey.

Appendix Table 2. Adjusted rates of respondent-rated poor health by education categories

Data name and population subgroup	Adjusted rates by educational attainment[1]				
	Not high school graduate, % (95% CI)	High school graduate, % (95% CI)	Some college, % (95% CI)	College graduate, % (95% CI)	Trend, *P*
Behavioral Risk Factor Surveillance System					
All (25-64 y)	70.0 (69.0, 71.1)	54.3 (53.7, 55.0)	46.4 (45.8, 47.0)	30.9 (30.5, 31.4)	<.001
NH Black (25-64 y)	72.7 (69.8, 75.5)	60.9 (59.2, 62.7)	52.5 (50.7, 54.3)	38.7 (37.0, 40.3)	<.001
Hispanic (25-64 y)	74.5 (72.8, 76.2)	60.1 (58.2, 62.1)	48.6 (46.4, 50.7)	36.4 (34.7, 38.1)	<.001
NH White (25-64 y)	67.9 (66.3, 69.4)	50.8 (50.1, 51.5)	43.8 (43.1, 44.4)	27.2 (26.8, 27.6)	<.001
NH Asian (25-64 y)	63.2 (51.1, 75.3)	52.9 (47.2, 58.6)	47.0 (41.4, 52.6)	35.3 (33.1, 37.5)	<.001
All (≥ 65 y)	79.3 (78.0, 80.5)	63.4 (62.2, 64.2)	56.2 (55.4, 57.0)	42.2 (41.5, 42.9)	<.001
NH Black (≥ 65 y)	78.4 (75.1, 81.8)	76.6 (74.3, 78.9)	72.1 (69.0, 75.2)	54.2 (50.9, 57.7)	<.001
Hispanic (≥ 65 y)	85.9 (83.2, 88.5)	71.1 (67.1, 75.2)	67.0 (62.4, 71.6)	52.6 (48.3, 56.8)	<.001
NH White (≥ 65 y)	75.6 (74.1, 77.2)	60.8 (59.9, 61.7)	53.7 (52.9, 54.5)	39.8 (39.1, 40.5)	<.001
NH Asian (≥ 65 y)	73.3 (55.7, 91.0)	70.2 (59.5, 80.9)	59.3 (48.0, 70.7)	51.2 (44.4, 58.0)	.002
National Health Interview Survey					
All (2-17 y)	18.0 (15.8, 20.2)	18.2 (16.9, 19.4)	14.6 (13.7, 15.6)	10.4 (9.6, 11.1)	<.001
NH Black (2-17 y)	28.4 (22.0, 34.7)	24.2 (20.9, 27.6)	20.8 (18.3, 23.3)	15.7 (12.8, 18.6)	<.001
Hispanic (2-17 y)	22.0 (18.8, 25.2)	23.1 (20.5, 25.8)	20.0 (17.7, 22.4)	15.4 (12.8, 17.9)	<.001
NH White (2-17 y)	17.0 (12.5, 21.4)	15.3 (13.4, 17.2)	11.2 (10.2, 12.2)	7.4 (6.7, 8.0)	<.001
NH Asian (2-17 y)	13.3 (4.0, 22.7)	15.3 (8.9, 21.7)	12.0 (7.9, 16.2)	11.0 (9.0, 13.0)	.010
All (25-64 y)	48.7 (47.3, 50.2)	43.6 (42.7, 44.4)	38.5 (37.8, 39.3)	25.6 (25.0, 26.3)	<.001
NH Black (25-64 y)	58.8 (55.6, 62.1)	51.5 (49.3, 53.6)	44.9 (42.9, 46.9)	35.2 (32.8, 37.7)	<.001
Hispanic (25-64 y)	45.9 (43.8, 48.1)	44.3 (42.2, 46.4)	42.4 (40.3, 44.5)	42.4 (40.3, 44.5)	<.001
NH White (25-64 y)	52.8 (50.7, 54.9)	42.3 (41.2, 43.2)	36.6 (35.8, 37.4)	23.3 (22.6, 23.9)	<.001
NH Asian (25-64 y)	45.3 (40.0, 50.5)	36.7 (33.1, 40.4)	33.6 (30.5, 36.6)	24.6 (22.9, 26.3)	<.001
All (≥ 65 y)	67.1 (65.5, 68.6)	59.2 (58.2, 60.3)	54.8 (53.8, 55.9)	44.3 (43.1, 45.5)	<.001
NH Black (≥ 65 y)	77.9 (74.9, 81.0)	72.9 (70.1, 75.7)	65.4 (61.7, 69.1)	62.6 (57.7, 67.6)	<.001
Hispanic (≥ 65 y)	75.5 (72.8, 78.3)	66.2 (62.0, 70.5)	65.1 (59.8, 70.4)	58.5 (52.8, 64.3)	<.001
NH White (≥ 65 y)	64.0 (62.0, 66.1)	56.3 (55.2, 57.6)	51.8 (50.6, 53.0)	40.4 (39.1, 41.7)	<.001
NH Asian (≥ 65 y)	72.7 (66.7, 78.7)	64.3 (58.4, 70.2)	61.0 (54.0, 68.0)	52.8 (48.4, 57.2)	<.001
National Health and Nutrition Examination Survey					
All (25-64 y)	70.7 (63.9, 77.6)	64.0 (61.2, 66.8)	53.8 (50.4, 57.3)	38.8 (34.0, 43.6)	<.001

NH Black (25-64 y)	69.0 (60.6, 77.4)	61.8 (55.7, 67.9)	58.5 (54.9, 62.1)	51.9 (45.2, 58.7)	.004
Hispanic (25-64 y)	78.4 (73.7, 83.2)	73.8 (67.6, 79.9)	60.5 (54.6, 66.4)	45.8 (38.0, 53.5)	<.001
NH White (25-64 y)	71.8 (58.1, 85.6)	63.1 (58.3, 67.9)	51.3 (46.3, 56.6)	34.5 (27.7, 41.4)	<.001
All (≥ 65 y)	82.6 (77.5, 87.7)	66.0 (59.8, 72.2)	58.8 (50.7, 66.8)	43.8 (37.7, 50.0)	<.001
NH Black (≥ 65 y)	88.6 (82.1, 93.1)	76.9 (67.5, 86.3)	84.3 (74.5, 94.1)	73.3 (62.3, 84.3)	.088
NH White (≥ 65 y)	78.8 (71.7, 85.9)	64.2 (56.2, 72.2)	53.7 (43.8, 63.5)	39.0 (32.0, 46.0)	<.001
Health & Retirement Study (≥ 65 y)					
All	75.0 (72.0, 78.0)	66.9 (65.0, 68.7)	61.1 (58.3, 64.0)	52.0 (50.0, 54.0)	<.001
NH Black	81.9 (78.3, 85.5)	75.9 (70.3, 81.6)	71.7 (65.1, 78.4)	67.0 (58.7, 75.4)	<.001
Hispanic	84.2 (80.8, 87.7)	72.3 (67.4, 77.1)	71.8 (66.5, 77.1)	63.7 (56.4, 71.0)	<.001
NH White	72.1 (68.4, 75.8)	61.5 (59.5, 63.5)	55.5 (53.2, 57.8)	44.1 (41.9, 46.2)	<.001

Note. NH indicates non-Hispanic. CI indicates confidence interval.

Source. Our data from the National Health Interview Study was collected in the US in 2015-2018. Data from the National Health and Nutrition Examination Survey was collected in the US in 2017-March 2020. Data from the Health and Retirement Study was collected in the US in 2016. Data from the Behavioral Risk Factor Surveillance System was collected in the US in 2016-2020.

[1] Age, sex, race/ethnicity, and education level were adjusted in the full sample. In race/ethnicity-stratified sample, age, sex, and income level were adjusted. As an exception, income was not adjusted among children and middle-aged adults in the Behavioral Risk Factor Surveillance System (BRFSS), and age and income were not adjusted among older adults in the BRFSS.

Appendix Table 3. Adjusted obesity rates by income categories

Data name and population subgroup	Adjusted rates by family income as percentage of FPL[1]				
	< 100% FPL, % (95% CI)	100-199% FPL, % (95% CI)	200-299% FPL, % (95% CI)	≥ 300% FPL, % (95% CI)	Trend, *P*
National Health Interview Survey					
All (2-17 y)	20.2 (17.5, 23.0)	16.4 (14.5, 18.2)	15.2 (13.3, 17.1)	11.5 (10.4, 12.5)	<.001
NH Black (2-17 y)	23.8 (18.2, 29.4)	20.0 (15.3, 24.7)	19.9 (14.0, 25.8)	16.2 (11.8, 20.6)	<.001
Hispanic (2-17 y)	24.3 (19.7, 28.8)	19.7 (16.1, 23.3)	16.2 (11.9, 20.5)	13.8 (10.6, 17.1)	<.001
NH White (2-17 y)	19.0 (14.7, 23.4)	15.6 (12.7, 18.6)	13.4 (10.9, 15.9)	9.7 (8.6, 10.8)	<.001
NH Asian (2-17 y)	15.4 (4.8, 25.9)	5.7 (1.0, 10.4)	28.2 (15.4, 41.1)	10.3 (7.0, 13.7)	.003
All (25-64 y)	37.4 (36.0, 38.8)	36.3 (35.1, 37.5)	35.6 (34.4, 36.8)	32.8 (32.1, 33.5)	<.001
NH Black (25-64 y)	43.2 (40.3, 46.1)	45.8 (43.0, 48.7)	46.0 (42.6, 49.4)	47.9 (45.3, 50.4)	.058
Hispanic (25-64 y)	41.6 (38.9, 44.3)	37.2 (34.7, 39.8)	37.5 (34.5, 40.4)	33.4 (31.3, 35.5)	<.001
NH White (25-64 y)	37.9 (36.0, 39.8)	36.9 (35.3, 38.5)	35.5 (33.9, 37.2)	32.1 (31.3, 32.9)	<.001
NH Asian (25-64 y)	15.4 (10.1, 20.6)	15.1 (10.9, 19.3)	10.5 (6.7, 14.3)	12.5 (10.9, 14.1)	.190
All (≥ 65 y)	31.7 (29.7, 33.8)	30.2 (28.8, 31.7)	30.6 (29.0, 32.2)	28.7 (27.7, 29.7)	.009
NH Black (≥ 65 y)	41.5 (36.6, 46.4)	37.9 (33.8, 42.0)	43.5 (37.5, 49.5)	37.8 (33.4, 42.2)	.538
Hispanic (≥ 65 y)	37.9 (32.0, 43.8)	30.4 (26.2, 34.7)	33.0 (26.3, 39.7)	27.0 (22.1, 31.8)	.025
NH White (≥ 65 y)	29.9 (26.9, 32.8)	30.6 (29.0, 32.3)	30.3 (28.6, 32.0)	28.7 (27.7, 29.7)	.057
NH Asian (≥ 65 y)	10.4 (4.7, 16.2)	10.7 (5.2, 16.2)	8.6 (3.1, 14.1)	11.7 (8.1, 15.3)	.751
National Health and Nutrition Examination Survey					
All (2-17 y)	22.5 (18.7, 26.2)	24.6 (19.8, 29.4)	15.6 (12.6, 18.5)	13.5 (10.6, 16.5)	<.001
NH Black (2-17 y)	20.9 (18.2, 23.7)	26.9 (22.2, 31.7)	24.3 (15.8, 32.8)	18.1 (7.9, 28.2)	<.001
Hispanic (2-17 y)	29.3 (21.4, 37.2)	25.8 (16.1, 35.4)	20.0 (11.7, 28.2)	19.4 (13.2, 25.6)	.122
NH White (2-17 y)	21.1 (15.3, 26.9)	25.4 (19.0, 31.9)	12.7 (8.8, 16.6)	11.6 (8.3, 14.8)	.001
All (25-64 y)	42.3 (37.5, 47.2)	39.4 (34.5, 44.3)	49.5 (44.5, 54.5)	43.1 (38.0, 48.1)	.584
NH Black (25-64 y)	51.8 (46.7, 56.9)	53.5 (47.3, 59.8)	49.8 (41.9, 57.7)	57.0 (50.7, 63.3)	.306
Hispanic (25-64 y)	45.8 (37.2, 54.5)	48.7 (41.3, 56.0)	46.6 (36.6, 56.7)	49.4 (40.7, 58.2)	.687
NH White (25-64 y)	44.0 (37.2, 50.8)	36.9 (29.5, 44.3)	54.8 (48.0, 61.6)	42.8 (35.9, 49.7)	.835
All (≥ 65 y)	37.6 (27.4, 47.9)	40.7 (33.9, 47.5)	40.2 (31.8, 48.6)	42.2 (34.8, 49.6)	.641
NH Black (≥ 65 y)	42.4 (27.8, 57.0)	43.2 (33.7, 52.6)	44.5 (30.3, 58.7)	54.9 (45.6, 64.2)	.100
NH White (≥ 65 y)	39.2 (23.3, 55.0)	42.1 (33.4, 50.9)	41.6 (31.5, 51.6)	42.9 (34.9, 51.0)	.823
Health & Retirement Study (≥ 60 y)					

All	27.4 (23.1, 31.7)	31.6 (28.4, 34.7)	32.2 (29.7, 34.6)	29.2 (27.5, 30.9)	.680
NH Black	37.5 (31.5, 43.6)	33.6 (26.0, 41.3)	36.4 (30.0, 42.9)	42.8 (36.9, 48.7)	.246
Hispanic	39.2 (32.3, 46.1)	47.5 (42.9, 52.1)	37.1 (32.4, 41.7)	38.2 (33.2, 43.2)	.451
NH White	29.6 (26.0, 33.1)	35.5 (31.8, 39.2)	36.8 (34.1, 39.5)	33.6 (32.3, 34.8)	.656

Note. NH indicates non-Hispanic. CI indicates confidence interval.

Source. Our data from the National Health Interview Study was collected in the US in 2015-2018. Data from the National Health and Nutrition Examination Survey was collected in the US in 2017-March 2020. Data from the Health and Retirement Study was collected in the US in 2016.

[1] Age, sex, race/ethnicity, and education level were adjusted in the full sample. In race/ethnicity-stratified sample, age, sex, and education level were adjusted. As an exception, education was not adjusted in the National Health and Nutrition Examination Survey.

Appendix Table 4. Adjusted obesity rates by education categories

Data name and population subgroup	Adjusted rates by educational attainment[1]				
	Not high school graduate, % (95% CI)	High school graduate, % (95% CI)	Some college, % (95% CI)	College graduate, % (95% CI)	Trend, *P*
Behavioral Risk Factor Surveillance System					
All (25-64 y)	38.9 (37.7, 40.1)	38.1 (37.4, 38.7)	37.2 (36.6, 37.8)	27.1 (26.7, 27.5)	<.001
NH Black (25-64 y)	45.4 (42.2, 48.6)	47.1 (45.4, 48.8)	46.8 (45.0, 48.6)	39.7 (38.1, 41.3)	<.001
Hispanic (25-64 y)	42.9 (40.9, 45.0)	39.9 (38.0, 41.8)	39.6 (37.5, 41.7)	28.9 (27.4, 30.4)	<.001
NH White (25-64 y)	37.7 (36.2, 39.2)	37.7 (37.0, 38.4)	36.9 (36.2, 37.5)	26.4 (26.0, 26.8)	<.001
NH Asian (25-64 y)	20.1 (9.8, 30.4)	22.2 (16.9, 27.4)	18.2 (14.1, 22.2)	10.1 (8.8, 11.4)	<.001
All (≥ 65 y)	33.0 (31.4, 34.5)	31.2 (30.4, 31.9)	30.7 (30.0, 31.4)	23.8 (23.2, 24.4)	<.001
NH Black (≥ 65 y)	39.1 (35.4, 42.7)	36.5 (33.8, 39.2)	35.8 (32.7, 38.9)	31.5 (28.4, 34.5)	.003
Hispanic (≥ 65 y)	34.4 (30.7, 38.2)	32.4 (28.1, 36.6)	32.4 (27.6, 37.1)	27.7 (23.8, 31.5)	.052
NH White (≥ 65 y)	31.5 (29.8, 33.1)	30.7 (29.9, 31.5)	30.5 (29.8, 31.3)	23.6 (23.1, 24.2)	<.001
NH Asian (≥ 65 y)	8.1 (2.5, 13.6)	6.6 (3.2, 10.0)	8.3 (3.6, 13.0)	8.3 (5.3, 11.3)	.635
National Health Interview Survey					
All (2-17 y)	19.2 (16.0, 22.4)	17.7 (15.6, 19.7)	17.2 (15.7, 18.8)	10.4 (9.3, 15.5)	<.001
NH Black (2-17 y)	28.2 (17.4, 39.1)	24.0 (17.8, 30.2)	21.9 (17.8, 25.9)	13.4 (9.7, 17.2)	<.000
Hispanic (2-17 y)	22.9 (18.4, 27.5)	23.3 (19.0, 27.7)	18.1 (15.0, 21.1)	12.1 (8.9, 15.2)	<.001
NH White (2-17 y)	14.2 (8.6, 19.8)	12.8 (10.3, 15.4)	16.0 (14.0, 18.0)	9.2 (7.9, 10.4)	<.001
NH Asian (2-17 y)	11.1 (0.0, 23.5)	15.4 (3.9, 26.9)	16.6 (9.1, 24.0)	9.4 (6.2, 12.6)	.003
All (25-64 y)	38.4 (36.8, 39.9)	38.0 (37.0, 39.0)	39.1 (38.1, 40.1)	27.9 (27.1, 28.7)	<.001
NH Black (25-64 y)	51.2 (47.1, 55.4)	48.6 (46.0, 51.2)	47.9 (45.2, 50.6)	38.4 (35.6, 41.2)	<.001
Hispanic (25-64 y)	37.9 (35.4, 40.4)	35.2 (32.4, 37.9)	41.9 (39.6, 44.2)	31.2 (28.6, 33.8)	.077
NH White (25-64 y)	38.9 (36.5, 41.2)	38.9 (37.6, 40.2)	39.0 (37.9, 40.1)	27.1 (26.2, 28.0)	<.001
NH Asian (25-64 y)	14.4 (8.5, 20.2)	14.7 (10.1, 19.2)	17.3 (13.4, 21.2)	11.5 (9.8, 13.1)	.096
All (≥ 65 y)	33.7 (31.9, 35.5)	32.3 (31.1, 33.5)	31.5 (30.2, 32.8)	23.8 (22.6, 25.1)	<.001
NH Black (≥ 65 y)	39.5 (34.8, 44.2)	44.8 (40.7, 48.8)	39.4 (34.8, 43.9)	32.7 (27.3, 38.0)	.052
Hispanic (≥ 65 y)	33.1 (29.3, 36.8)	34.5 (28.7, 40.4)	29.5 (23.5, 35.6)	26.9 (19.7, 34.1)	.114
NH White (≥ 65 y)	34.8 (32.3, 37.2)	32.0 (30.6, 33.3)	31.9 (30.6, 33.3)	23.5 (22.3, 24.8)	<.001
NH Asian (≥ 65 y)	17.3 (10.1, 24.4)	13.1 (7.7, 18.5)	10.6 (4.6, 16.6)	7.8 (4.7, 10.9)	.012
National Health and Nutrition Examination Survey					
All (25-64 y)	44.7 (38.8, 50.5)	46.6 (40.2, 53.0)	47.9 (43.3, 52.5)	36.3 (32.9, 39.8)	.001

NH Black (25-64 y)	53.0 (41.0, 65.0)	52.8 (45.7, 59.9)	54.4 (50.2, 58.6)	54.1 (47.2, 61.0)	.641
Hispanic (25-64 y)	49.4 (42.8, 56.0)	52.5 (44.2, 60.8)	49.8 (43.7, 56.0)	38.0 (30.8, 45.2)	.116
NH White (25-64 y)	44.6 (31.3, 57.9)	47.9 (37.9, 57.8)	49.0 (43.0, 55.0)	36.1 (30.8, 41.5)	.005
All (≥ 65 y)	43.0 (35.2, 50.8)	47.5 (41.5, 53.5)	42.5 (35.1, 50.0)	33.8 (24.6, 43.0)	.047
NH Black (≥ 65 y)	53.5 (42.6, 64.4)	49.2 (42.1, 56.4)	42.2 (33.4, 51.1)	45.5 (29.0, 61.9)	.413
NH White (≥ 65 y)	48.3 (35.8, 60.7)	49.7 (43.0, 56.4)	43.3 (35.2, 51.4)	34.2 (23.3, 45.1)	.027
Health & Retirement Study (age ≥ 65 y)					
All	34.2 (31.0, 37.4)	32.7 (30.2, 35.2)	31.2 (29.0, 33.3)	24.2 (22.2, 26.3)	<.001
NH Black	41.7 (35.6, 47.8)	35.4 (30.7, 40.2)	40.6 (34.2, 46.9)	34.1 (26.7, 41.4)	.381
Hispanic	41.0 (37.4, 44.5)	37.4 (31.6, 43.2)	43.8 (37.5, 50.0)	39.3 (30.8, 47.7)	1.000
NH White	38.2 (35.1, 41.4)	38.1 (35.8, 40.3)	35.3 (33.4, 37.2)	28.8 (26.7, 31.0)	<.001

Note. NH indicates non-Hispanic. CI indicates confidence interval.

Source. Our data from the National Health Interview Study was collected in the US in 2015-2018. Data from the National Health and Nutrition Examination Survey was collected in the US in 2017-March 2020. Data from the Health and Retirement Study was collected in the US in 2016. Data from the Behavioral Risk Factor Surveillance System was collected in the US in 2016-2020.

[1] Age, sex, race/ethnicity, and education level were adjusted in the full sample. In race/ethnicity-stratified sample, age, sex, and income level were adjusted. As an exception, income was not adjusted among children and middle-aged adults in the Behavioral Risk Factor Surveillance System (BRFSS), and age and income were not adjusted among older adults in the BRFSS.
